# Predictive accuracy of serum IL-6, IL-17 and sST2 levels for diuretic resistance in patients with chronic heart failure

**DOI:** 10.1186/s12872-026-05756-2

**Published:** 2026-04-13

**Authors:** Dingjun Dong, Cong Sun, Bin Li, Dongdong Huang, Wanyao Zhang, Ming Lu

**Affiliations:** 1 Department of Cardiovascular Medicine, Xiangyang Central Hospital Affiliated Hospital to Hubei University of Arts and Science, 136 Jingzhou Street, Xiangcheng District, Xiangyang City, 441000 Hubei Province China; 2 College of Medical, Wuhan University of Science and Technology, Wuhan City, Hubei Province China

**Keywords:** IL-6, IL-17, sST2, Chronic heart failure, Clinical diuretic strategies, Diuretic resistance

## Abstract

**Objective:**

To determine the predictive value of serum levels of Interleukin-6 (IL-6), interleukin-17 (IL-17), soluble suppression of tumorigenicity 2 protein (sST2) for diuretic resistance in patients with chronic heart failure (CHF).

**Methods:**

His retrospective cohort study enrolled patients with CHF patients treated at Xiangyang Central Hospital between July 2019 and July 2022. Participants were categorized into two groups: the diuretic resistance (DR) group (*n* = 70) and non-DR group (*n* = 80). Diuretic resistance was established by a urine output < 0.5–1.0 mL/kg/h following the administration of ≥ 80 mg of furosemide. General clinical data and serum indexes were compared between groups. Multivariate logistic regression identified independent DR risk factors. Bootstrap resampling (1000 replicates) was used to estimate 95% confidence intervals for the area under the curve, sensitivity, and specificity in receiver operating characteristic analysis.

**Results:**

Baseline characteristics, including age, sex, and comorbidities, were comparable between the diuretic resistance (DR) and non-DR groups (all *P* > 0.05). Compared with the non-DR group, patients with DR exhibited significantly lower urine output and urinary sodium concentration, alongside impaired renal function (higher Cr and lower eGFR), hypoalbuminemia, and elevated liver enzymes (ALT) (all *P* < 0.001). Furthermore, inflammatory markers (WBC, IL-6, IL-17, sST2) and cardiac dysfunction indicators (NT-proBNP, LVEDd, LAVI, and TRPG) were significantly elevated in the DR group (all *P* < 0.05). After adjusting for age, LVEF, and eGFR in the multivariate logistic regression, IL-6 (OR: 1.860 per 10 pg/mL), IL-17 (OR: 1.952 per 10 pg/mL), and sST2 (OR: 1.450 per 10 µg/L) remained independently associated with the risk of diuretic resistance (all *P* < 0.05). ROC curves analysis revealed that combined assessment of IL-6, IL-17 and sST2 provided the highest predictive accuracy for diuretic resistance, with an AUC of 0.861 (95% bootstrapped CI: 0.795–0.912), a sensitivity of 81.43%, and a specificity of 86.25%, outperforming individual biomarkers (*P* = 0.0001).

**Conclusion:**

Serum IL-6, IL-17, and sST2 levels are associated with diuretic resistance in hospitalized patients with chronic heart failure. The integration of these biomarkers may provide enhanced predictive value for identifying diuretic resistance compared to individual assessments.

**Supplementary Information:**

The online version contains supplementary material available at 10.1186/s12872-026-05756-2.

## Highlight

• Serum IL-6, IL-17, and sST2 levels serve as predictive markers for diuretic resistance in patients with chronic heart failure.

• Patients with diuretic resistance exhibit elevated IL-6, IL-17, sST2, and NT-proBNP levels.

• Left ventricular function parameters (LVEF and LVEDd) and NtproBNP are notably higher in diuretic-resistant patients.

**Supplementary Information:**

The online version contains supplementary material available at 10.1186/s12872-026-05756-2.

## Introduction

Chronic heart failure (CHF) is a leading cause of morbidity and mortality globally and the absolute number of the patients with CHF is increasing as a result of the increasing number of older individuals [1]. 2022 AHA/ACC/HFSA guideline for the management of heart failure (HF) is based on four evidence-supported pharmacological pillars: renin-angiotensin-aldosterone system inhibitors; beta-blockers; mineralocorticoid receptor antagonists (MRA); and sodium-glucose cotransporter 2 inhibitors (SGLT2i) [2]. Diuretics rapidly optimize hemodynamic parameters and relieve congestive symptoms but fail to modify the underlying pathological process of the disease. In contrast, ACEi and ARBs not only alleviate clinical symptoms but also slow disease progression via their cardioprotective effects, thus serving as essential pharmacotherapies for the management of chronic heart failure [3]. However, one-third of CHF patients develop diuretic resistance, and a large proportion requires high dosages to improve hypervolemic conditions [4]. Diuretic resistance is a major contributor to recurrent hospitalizations and mortality in patients with CHF, yet its recognition remains challenging due to the lack of precise diagnostic criteria.

Diuretic resistance is defined as the failure to achieve adequate renal excretion of water and sodium (Na⁺) to relieve volume overload, edema, or congestion despite the administration of a full therapeutic dose of a loop diuretic. Quantitatively, its definitions include a failure of oral furosemide (160 mg twice daily or equivalent) to increase Na^+^ excretion by at least 90 mmol over 3 days [5]. Alternatively, a spot urine sodium concentration of < 50 mmol obtained 1–2 h after loop diuretic administration is also indicative of diuretic resistance [6]. Multiple mechanisms contribute to the development of diuretic resistance in patients with CHF. From a pharmacokinetic perspective, the variable bioavailability of furosemide and the short duration of action of loop diuretics allow renal sodium reabsorption between doses. Pathophysiologically, excessive dietary sodium intake, electrolyte disturbances, and activation of renal sympathetic nerves further impair natriuretic response. At the nephron level, tubular adaptation—including enhanced proximal and distal sodium reabsorption—reduces diuretic efficacy and promotes tolerance over time.

Early and accurate identification of patients at risk of developing diuretic resistance, followed by timely and effective intervention, is essential to improve clinical outcome [5]. A high-throughput proteomic analysis specifically designed to profile patients with acute decompensated heart failure and to identify protein markers associated with rehospitalization, mortality, or diuretic response revealed that three proteins—matrix metalloproteinase-7 (MMP7), peptidoglycan recognition protein 1 (PGLYRP1), and renin (REN)—were significantly linked to a reduced diuretic response. Urinary MMP7, a biomarker of kidney injury, may indicate that chronic kidney disease (CKD) and heart failure share multiple physiological pathways and common risk factors [7]. Furthermore, Guo L et al. [8] reported that hemodynamic alterations in heart failure activate the sympathetic nervous system and the renin–angiotensin system, triggering systemic inflammation that further impairs cardiac function, thereby creating a self-perpetuating vicious cycle.

Emerging evidence highlights the roles of IL-6, IL-17, and sST2 in mediating DR in CHF, primarily through regulating inflammation and renal function. Animal studies demonstrate that IL-6 overexpression induces hypertensive concentric cardiac hypertrophy in mice, causing abnormal ventricular filling, reduced renal perfusion (impairing diuretic sensitivity), and activated inflammatory pathways that disrupt renal water-sodium excretion and worsen DR [9, 10]. In isoprenaline-induced CHF models, administration of anti-IL-17 neutralizing antibodies reduces myocardial collagen deposition by inhibiting the expression of matrix metalloproteinase 1 (MMP-1); conversely, excessive fibrosis driven by IL-17 signaling impairs cardiac circulatory function and diminishes renal sensitivity to diuretics—providing mechanistic evidence that IL-17 modulates DR in CHF through the regulation of myocardial and renal fibrotic processes [11–13]. Clinical data demonstrate elevated sST2 correlates with BNP and galectin-3 (CHF adverse outcome predictors); heightened sST2 activity drives myocardial fibrosis/inflammation, causing renal congestion/perfusion loss and reduced diuretic responsiveness. Integrating sST2 into multi-biomarker panels also improves CHF prognostic precision and guides DR assessment [14, 15].

The main objectives of this study are as follows: to investigate whether the combined detection of serum IL-6, IL-17, and sST2 levels can effectively predict the likelihood of diuretic resistance in patients with CHF; and to explore the potential value of this combined detection method in providing scientific evidence for formulating clinical diuretic therapy strategies for CHF patients, thereby optimizing the management of diuretic resistance in such patients.

## Methods

### Study design and participants

A total of 150 patients with chronic heart failure who received treatment in the Department of Cardiovascular Medicine at our hospital between July 2019 and July 2022 were included in this single-center retrospective study. In accordance with the Chinese Expert Consensus on the Diagnosis and Management of Diuretic Resistance in Patients with Heart Failure, the diagnostic criteria for diuretic resistance are established as follows: a urine output of < 0.5–1.0 mL/kg/h is observed in patients administered a furosemide dose of no less than 80 mg [16]. Based on the diagnostic criteria for diuretic resistance, the patients were classified into two groups: the diuretic resistance group (*n* = 70) and the non-diuretic resistance group (*n* = 80). Dingjun Dong and Cong Sun calculated total urine output of patients in CHF.

Inclusion criteria: (1) All participants meet the diagnostic criteria for chronic heart failure (CHF) specified in the 2018 Chinese Guidelines for the Diagnosis and Treatment of Heart Failure [17], presenting typical symptoms including fatigue, decreased exercise tolerance, persistent edema, chest tightness, shortness of breath, or orthopnea, with NT-proBNP levels exceeding 125 pg/mL and relevant abnormalities in auxiliary examinations. (2) Patients must have been classified as New York Heart Association functional class III or IV, with clinical evidence of cardiac dysfunction (including reduced or mid-range ejection fraction) [18]; (3) Patients with complete data.

Exclusion criteria: (1) Individuals were diagnosed with systemic malignant tumors or immune system disorders; (2) Stroke or acute liver failure within the last 6 months; (3) Individuals have experienced severe cognitive impairment that hinders their ability to participate in the study; (4) Known chronic liver failure with alanine aminotransferase or aspartate aminotransferase at five times the upper normal range; (5) Bladder dysfunction, incontinence, or inability to comply with timed urine.

This study was approved by the Ethics Committee of Xiangyang Central Hospital in accordance with regulatory and ethical guidelines pertaining to retrospective research studies (Ethical approval No. 2023-043) on 11th April 2023. Informed consent was waived for this retrospective study due to the exclusive use of de-identified patient data, which posed no potential harm or impact on patient care.

### Data collection

General clinical data were extracted from electronic medical records upon admission. Blood samples were collected on hospital day 1 (within 24 h of admission) to measure serum levels of IL-6, IL-17, sST2, and BNP using standard laboratory methods. A comprehensive echocardiographic examination was performed on hospital day 2–3 (median: day 2) to assess cardiac structure and function in accordance with Xiangyang Central Hospital’s clinical practice. DR was diagnosed on hospital day 5–7 based on predefined criteria, with all aforementioned examinations completed prior to DR confirmation.

### Laboratory parameters

Upon admission, 5 ml of venous blood was collected from each patient via the median cubital vein using a red-top vacuum blood collection tube. Subsequently, the blood sample was centrifuged at 3000r/min for 10 min using a high-speed centrifuge, and the supernatant was transferred to a sterile test tube and stored at -20 ℃ in a refrigerated cabinet.

HA3100 Hematology Analyzer (Kangtai Medical System (Qinhuangdao) Co., Ltd., China) used to quantify white blood cell count (WBC); A AU5800 Fully Automatic Biochemical Analyser (Beckman, USA) was employed to assess the liver function [Aspartate aminotransferase (AST), alanine aminotransferase (ALT)], renal function[creatinine (Cr), Blood Urea Nitrogen (BUN)] and lipid profiles [low-density lipoprotein cholesterol (LDL-C), high-density lipoprotein cholesterol (HDL-C) total cholesterol (TC), and triglycerides (TG)] using reagents supplied by Beijing Inspection-Xinchuangyuan Biotechnology Co., Ltd.

Inflammatory markers, including interleukin-6 (IL-6; Signalway Antibody, USA), interleukin-17 (IL-17; BioLegend, USA), high-sensitivity C-reactive protein (Hs-CRP; eBioscience, USA), N-terminal pro-B-type natriuretic peptide (NT-proBNP; EK-Bioscience, China), soluble suppression of tumorigenicity 2 (sST2; Tanda, China), and serum uric acid (UA; Rothen Medizinische Laboratorien AG, Basel, Switzerland), were quantified using enzyme-linked immunosorbent assay (ELISA) kits.

### Echocardiography examination

Echocardiography was performed using the GEVIVIDi/Viviq color echocardiography system (GE Healthcare, USA) to assess the dimensions of the left ventricle and atrium. The left ventricular end-diastolic diameter (LVEDd) was measured from the long-axis view of the left ventricle, while continuous Doppler measurements of mitral valve inflow velocity were obtained from the apical four-chamber view. The left ventricular ejection fraction (LVEF) was then calculated using Simpson’s biplane method.

### Other covariate measurements and definitions

Baseline characteristics were obtained form the electronic medical record before treatments and included age, sex, hypertension, type 2 diabetes mellitus and atrial fibrillation. The hypertension was defined as having a mean systolic blood pressure ≥ 140 mm Hg or diastolic blood pressure ≥ 90 mm Hg of the three screening visits [19]. The diagnosis of Type 2 diabetes mellitus was established according to the guidelines of the American Diabetes Association (ADA). Atrial fibrillation was diagnosed using a 12-lead electrocardiogram (ECG) interpreted by a specialist.

Total urine output was measured over the 12 h following loop diuretic administration (IV furosemide 612 ± 439 mg/day). In addition, urine sodium concentration was determined from a spot urine sample collected 2 h after administration.

Serum creatinine was measured enzymatically (Beijing Leadman). Estimated glomerular filtration rate (eGFR) was calculated via the Chinese modified MDRD formula (eGFR = 186×Scr⁻¹.¹⁵⁴×age⁻⁰.²⁰³×0.742 for females). Blood urea nitrogen (BUN; urease-glutamate dehydrogenase method, Shanghai Kehua), urine microalbumin/creatinine ratio (UACR; immunoturbidimetry, Roche Diagnostics), and 24-h urinary sodium excretion (ion-selective electrode method, Shenzhen Mindray) were also assessed.

### Statistical method

Statistical analyses were performed using SPSS 20.0 (IBM Corp., Armonk, NY, USA). Normally distributed continuous data were presented as mean ± SD and compared by independent-samples t-test; non-normally distributed data as median (IQR) with Mann-Whitney U test; categorical data as n (%) with χ² test. Multivariate logistic regression was used to identify risk factors for diuretic resistance in CHF patients, and binary ROC curve analysis with 1000 bootstrap replicates to evaluate predictive value and 95% CIs. Among 150 patients, the total missing data rate was 1.87% (4 values), restricted to IL-17 and sST2 due to instrument and sample issues, which were handled by MICE multiple imputation (5 datasets); sensitivity analysis verified no bias. A P value < 0.05 was considered statistically significant.

## Results

### Clinical data between the two groups

The key clinical characteristics of 70 patients with DR and 80 without non-DR are summarized in Table 1. Both groups exhibited comparable age distributions (58 ± 7 vs. 59 ± 8 years; *P* = 0.168), sex proportions (47% vs. 56% male; *P* = 0.265), and prevalence of comorbidities, including hypertension (80% vs. 83%; *P* = 0.551), type 2 diabetes mellitus (86% vs. 88%; *P* = 0.748), and atrial fibrillation (47% vs. 48%; *P* = 0.965). However, the DR group demonstrated significantly lower 12-hour total urine output (765 ± 182 mL vs. 2,140 ± 564 mL; *P* < 0.0001) and lower urine sodium concentration (69 ± 30 mmol/L vs. 111 ± 35 mmol/L; *P* < 0.0001) compared to the non-DR group.

**Table 1:** Comparison of clinical data and indexes between the two groups ($\overline{x}$ *± s*,* %*)

Item	Diuretic resistance group (*n* = 70)	Non-diuretic resistance group (*n* = 80)	χ^2^/t	*P* value
Age (years)	58 ± 7	59 ± 8	1.101	0.168
Sex (*n*[%])			1.241	0.265
Male	33 (47%)	45 (56%)		
female	37 (53%)	35 (44%)		
Hypertension (*n*[%])	56 (80%)	67 (83%)	0.356	0.551
Type 2 diabetes mellitus (*n*[%])	60 (86%)	70 (88%)	0.103	0.748
Atrial fibrillation (*n*[%])	33 (47%)	38 (48%)	0.002	0.965
Total urine output in past 12 h (mls)	765 ± 182	2140 ± 564	19.52	<0.0001
Urine sodium (mmol/L)	69 ± 30	111 ± 35	19.240	<0.0001

### Comparison of markers of liver and renal function between the two groups

As shown in Table 2, the DR group exhibited significantly higher ALT levels (33.98 ± 12.36 vs. 25.43 ± 6.60 U/L, *P* < 0.001). Regarding renal function, the DR group showed markedly impaired indicators compared with the non-DR group, characterized by significantly lower eGFR (67.50 ± 8.20 vs. 81.80 ± 9.40 mL/min/1.73m^2^, *P* < 0.0001) and higher serum creatinine (95.30 ± 14.20 vs. 78.60 ± 11.50 umol/L, *P* < 0.0001). Furthermore, the urine microalbumin/creatinine ratio was significantly higher, while 24-hour urinary sodium excretion and serum albumin were significantly lower in the DR group (all *P* < 0.001). Other parameters, including AST, BUN, and UA, showed no statistically significant differences between the two groups (*P* > 0.05).

**Table 2:** Comparison of markers of liver and renal function between the two groups ($\overline{x}\pm\mathrm{s}$)

Group	Diuretic resistance group (*n* = 70)	Non-diuretic resistance group (*n* = 80)	t	*P* value
AST (U/L)	32.96 ± 12.32	28.88 ± 11.59	2.088	0.385
ALT (U/L)	33.98 ± 12.36	25.43 ± 6.600	5.374	<0.0001
BUN (mmol/L)	6.68 ± 1.69	6.83 ± 1.63	0.552	0.581
Cr (µmol/L)	95.30 ± 14.20	78.60 ± 11.50	7.560	< 0.001
UA (µmol/L)	426.68 ± 172.42	423.59 ± 168.96	0.110	0.912
eGFR (mL/min/1.73 m²)	67.50 ± 8.20	81.80 ± 9.40	9.63	< 0.0001
Urine Microalbumin/Creatinine Ratio (mg/g)	108.50 ± 41.20	65.70 ± 29.30	8.24	< 0.0001
24-hour Urinary Sodium Excretion (mmol/24 h)	59.70 ± 21.80	92.40 ± 25.10	9.37	< 0.0001
Serum Albumin (g/L)	29.50 ± 3.80	34.00 ± 4.40	6.85	< 0.0001

*Abbreviations* *AST* Aspartate aminotransferase, *ALT* Alanine aminotransferase, *BUN* Blood Urea Nitrogen, *Cr* Serum Creatinine, *UA* Serum uric acid, *eGFR* Estimated Glomerular Filtration Rate

### Comparison of markers of inflammation between the two groups

The diuretic resistance group showed statistically significant elevations in several inflammatory and stress biomarkers. Specifically, their levels of WBC, IL-6, IL-17, and sST2 were significantly higher (*P*< 0.05) while the Hs-CRP levels between the two groups were similar (*P*> 0.05), as shown in Table 3.

**Table 3:** Comparison of markers of inflammation between the two groups ($\overline{x}\pm\mathrm{s}$)

Group	Diuretic resistance group (*n* = 70)	Non-diuretic resistance group (*n* = 80)	t	*P* value
WBC (10^9^/L)	6.42 ± 1.67	5.63 ± 1.98	2.621	0.009
IL-6 (pg/mL)	39.60 ± 15.60	32.61 ± 12.13	3.082	0.003
IL-17 (pg/mL)	42.44 ± 16.62	34.73 ± 13.38	3.145	0.002
sST2 (µg/L)	65.51 ± 22.38	58.87 ± 16.48	2.085	0.038
Hs-CRP (mg/L)	8.51 ± 7.94	7.70 ± 2.76	0.855	0.394

*Abbreviations* *IL-6* Interleukin-6, *IL-17* Interleukin-17, *sST2* Soluble suppression of tumorigenicity 2 protein, *WBC* White blood cell count, *Hs-CRP* High-sensitivity C-reactive protein

### Comparison of cardiac indexes between the two groups

Echocardiographic assessments followed the 2022 Chinese Guidelines for CHF and ASE recommendations. As summarized in Table 4, compared with the non-DR group, the DR group exhibited significantly larger cardiac dimensions, including LVEDd (55.01 ± 4.28 vs. 48.59 ± 3.91 mm, *P* < 0.0001) and LVESd (42.36 ± 3.85 vs. 35.72 ± 3.54 mm, *P* < 0.0001). Although the LVEF was numerically higher in the DR group (39.93% ± 5.12% vs. 29.63% ± 4.87%, *P* < 0.0001), these patients demonstrated more severe diastolic dysfunction, indicated by a higher E/A ratio, lower lateral e’, and increased LAVI (all *P* < 0.0001). Additionally, the TRPG was significantly elevated in the DR group (38.60 ± 6.20 vs. 26.30 ± 5.08 mmHg, *P* < 0.0001). Serum NT-proBNP levels were approximately fourfold higher in the DR group (5948.29 ± 1865.34 vs. 1568.34 ± 628.47 pg/mL, *P* < 0.0001), reflecting increased hemodynamic stress and more severe heart failure.

**Table 4:** Comparison of cardiac indexes between the two groups ($\overline{x}\pm\mathrm{s}$)

Group	Diuretic resistance group (*n* = 70)	Non-diuretic resistance group (*n* = 80)	t	*P* value
LVEDd (mm)	55.01 ± 4.28	48.59 ± 3.91	9.26	< 0.0001
LVESd ( mm)	42.36 ± 3.85	35.72 ± 3.54	11.83	< 0.0001
LVEF (%)	39.93 ± 5.12	29.63 ± 4.87	12.57	< 0.0001
E/A ratio	1.82 ± 0.35	1.24 ± 0.28	11.49	< 0.0001
e’(cm/s)	5.20 ± 1.10	7.80 ± 1.30	12.08	< 0.0001
LAVI ( mL/m²)	42.50 ± 5.30	34.20 ± 4.60	10.15	< 0.0001
TRPG (mmHg)	38.60 ± 6.20	26.30 ± 5.80	11.32	< 0.0001
NT-proBNP (pg/mL)	5948.29 ± 1865.34	1568.34 ± 628.47	18.74	< 0.0001

*Abbreviations* *LVEDd* Left ventricular end-diastolic diameter, *LVESd* Left Ventricular End-Systolic dimension, *LVEF* Left ventricular ejection fraction, *E/A ratio* Ratio of early to late diastolic transmitral flow velocity, *e’* Early diastolic mitral annular velocity, *LAVI* Left Atrial Volume Index, *TRPG* Tricuspid Regurgitation Pressure Gradient, *NT-proBNP* N-Terminal pro-B-type natriuretic peptide

### Multivariate logistic regression analysis of the influencing factors of diuretic resistance in patients with chronic heart failure

To identify independent predictors of DR in patients with CHF, a multivariate logistic regression model was constructed, incorporating inflammatory biomarkers (IL-6, IL-17, sST2) and clinical covariates (age, LVEF, and eGFR). The results, as detailed in Table 5, confirmed that IL-6 (OR: 1.860 per 10 pg/mL increase; 95% CI: 1.225–2.826; *P* = 0.001), IL-17 (OR: 1.952 per 10 pg/mL increase; 95% CI: 1.243–3.065; *P* = 0.001), and sST2 (OR: 1.450 per 10 ug/L increase; 95% CI: 1.010–2.082; *P* = 0.043) were all independent risk factors for DR. Among the clinical indicators, LVEF (OR: 1.096; *P* = 0.005) and eGFR (OR: 1.048; *P* = 0.014) also demonstrated significant independent associations with DR risk, whereas age showed no statistical significance (*P* = 0.605).

Collectively, these findings suggest that DR in CHF patients is a multifactorial condition driven by both systemic inflammation and impairment of cardiac and renal functions. The independent predictive value of IL-6, IL-17, and sST2, even after adjusting for heart failure severity and renal status, provides a robust theoretical basis for early clinical identification and risk stratification of high-risk populations.

**Table 5:** Logistic regression analysis of diuretic resistance in patients with chronic heart failure

Index	β	S	Wald	OR	95%CI	*P* value
IL-6 (per 10 pg/mL increase)	0.621	0.189	10.825	1.860	1.225 to 2.826	0.001
IL-17 (per 10 pg/mL increase)	0.668	0.201	11.156	1.952	1.243 to 3.065	0.001
sST2 (per 10 µg/L increase)	0.372	0.185	4.089	1.450	1.010 to 2.082	0.043
Age (years)	0.015	0.029	0.268	1.015	0.959 to 1.074	0.605
LVEF (%)	0.092	0.033	7.841	1.096	1.029 to 1.168	0.005
eGFR (mL/min/1.73 m²)	0.047	0.019	6.002	1.048	1.010 to 1.087	0.014

### ROC curve analysis

**Fig. 1:**
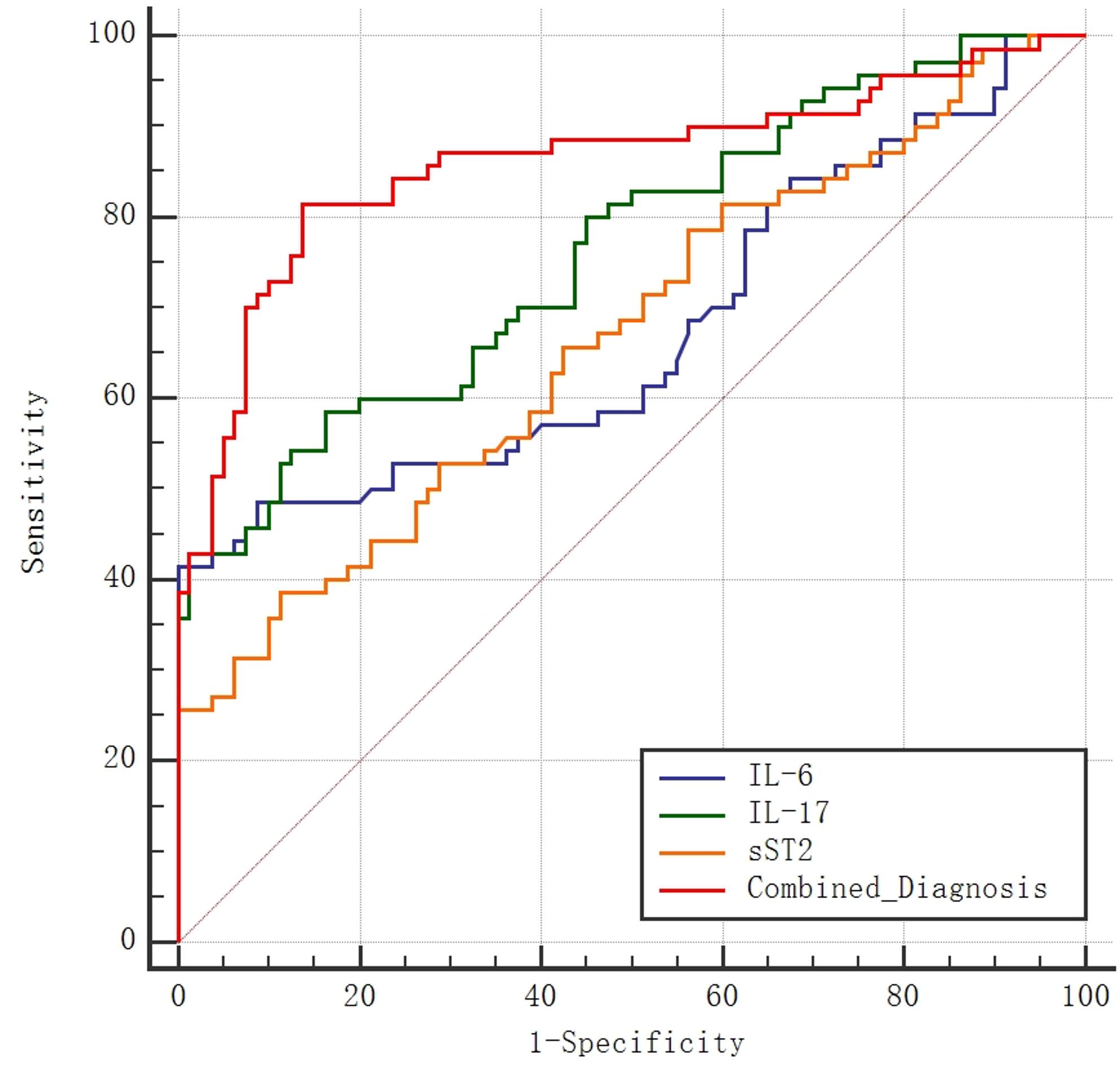
The ROC curve analysis was performed for IL-6, IL-17, and sST2 levels, as well as for their combined diagnostic performance. Abbreviations: Interleukin-6 (IL-6), interleukin-17 (IL-17), soluble suppression of tumorigenicity 2 protein (sST2)

The diagnostic performance of IL-6, IL-17, sST2, and their combined model was evaluated using ROC curve analysis (Table 6; Fig. 1). Among individual biomarkers, IL-17 demonstrated the highest area under the curve (AUC) of 0.764 (95% CI: 0.688–0.830, *P* < 0.0001), followed by IL-6 (AUC: 0.675; 95% CI: 0.594–0.750, *P* < 0.0001) and sST2 (AUC: 0.667; 95% CI: 0.586–0.742, *P* = 0.0002). Notably, the combined diagnostic model yielded superior predictive power, with an AUC of 0.861 (95% CI: 0.795–0.912, *P* = 0.0001). At the optimal cut-off value of 0.4871, the combined model achieved a sensitivity of 81.43% and a specificity of 86.25%. Bootstrap resampling (1000 replicates) confirmed the robustness of these estimates, yielding a consistent bootstrapped 95% CI of 0.795–0.912 for the combined model.

**Table 6:** ROC curve analysis of inflammatory biomarkers for predicting diuretic resistance

Index	AUC	95%CI (AUC)	*P*	cut-off	sensitivity	specificity	Youden Index
IL-6	0.675	0.594–0.750	< 0.0001	> 43.71	41.43%	90%	0.4143
IL-17	0.764	0.688–0.830	< 0.0001	> 42.94	58.57%	83.75%	0.4232
sST2	0.667	0.586–0.742	0.0002	> 70.44	38.57%	88.75%	0.2732
Combined diagnosis	0.861	0.795–0.912	0.0001	> 0.4871	81.43%	86.25%	0.6768

## Discussion

Inflammation is considered a key driver for both chronic and acute heart failure (AHF) decompensation, and it is associated with a poor prognosis. Proinflammatory activation in HF has multiple adverse effects, including worsening cardiac contractility, stimulation of fibrosis, renal and vascular dysfunction, fluid retention or redistribution, disruption of the endothelial barrier leading to increased capillary permeability, and neurohormonal activation—all of which may promote the development of congestion [20]. In this study, we observed that the levels of WBC, serum sST2, IL-6 and IL-17 were significantly higher in the diuretic resistance group than in the non-diuretic resistance group. A meta-analysis indicated that elevated C-reactive protein, increased neutrophil count, decreased lymphocyte count, higher red blood cell distribution width, lower platelet count, higher neutrophil-to-lymphocyte ratio, higher lymphocyte-to-monocyte ratio, elevated WBC, and higher systemic inflammation index were all associated with poor prognosis in patients with AHF [21]. In this study, larger LVEDd and elevated NT-proBNP levels were significantly associated with diuretic resistance, suggesting that these patients suffer from more advanced cardiac structural remodeling and overall clinical deterioration. Although the DR group exhibited a relatively higher LVEF, this should not be misinterpreted as superior cardiac performance. Instead, this paradox reflects a complex interplay of increased ventricular stiffness and elevated cardiac filling pressures, characteristic of heart failure with preserved or mid-range ejection fraction. Mechanistically, the marked elevation of TRPG and LAVI in the DR group points toward severe diastolic dysfunction and right-heart involvement. This leads to renal venous congestion, which increases intrarenal pressure and reduces the net glomerular filtration pressure gradient. Such hemodynamic congestion, rather than simple pump failure, impairs the delivery and efficacy of loop diuretics at the nephron level. Therefore, diuretic resistance in these patients is driven by high filling pressures and systemic venous congestion, which persist despite a compensated systolic ejection fraction, ultimately translating into a worse clinical prognosis.

A randomized, open-label pilot trial further showed that higher inflammatory markers (hsCRP > 20 mg/L, IL-6 > 13 pg/mL) correlated with greater relief of congestion in HF, highlighting inflammation’s key role [20, 22]. In this study, the multivariate logistic regression analysis identified elevated IL-6, IL-17, and sST2 levels as independent influencing factors of diuretic resistance in patients with CHF. The ROC analysis indicated combined assessment of IL-6, IL-7 and sST2 provided the highest sensitivity and specificity for predicting diuretic resistance in patients with CHF. Xu L et al. [23] reported that UA can cause vascular endothelial injury, thickening and hardening of the vascular wall, and then forming plaques, resulting in lumen stenosis, which indicated that body’s inflammatory response and promote platelet aggregation is an independent risk factor for cardiovascular disease. Among these markers, sST2 (IL-33 receptor) is considered an effective biomarker of adverse outcome risk stratification in HF patients [24]. IL-6 and IL-17 are common pro-inflammatory cytokines and important biochemical markers reflecting the systemic’s inflammatory response. Such inflammatory factors can cause renal endothelial vascular injury, aggravate renal ischemic damage, induce diuretic resistance, and ultimately worsen patient prognosis [25].

Type 2 diabetes is a key independent risk factor for heart failure, with insulin resistance driving cardiomyocyte metabolic dysfunction (impaired glucose utilization, excessive fatty acid accumulation) and subsequent mitochondrial damage, oxidative stress, and reduced cardiac contractility [26]. Persistent hyperglycemia in type 2 diabetes further exacerbates cardiac injury via advanced glycation end product (AGE) formation, myocardial inflammation/fibrosis, and SGLT2-mediated sodium reabsorption that causes fluid overload and blunted diuretic response, and it is thus rational to evaluate it as an independent risk factor in diuretic-related studies.

Furthermore, several other molecules and markers involved in the regulation of diuresis in heart failure, such as the renin–angiotensin–aldosterone system and endothelin-1, also play important roles. In a study of 211 patients with AHF, those with elevated RAAS activity (RAAS+/+) exhibited lower blood pressure, reduced serum sodium and urinary Na⁺ levels, required higher doses of furosemide at discharge, and had an increased risk of one-year mortality [27]. Another study involving 113 AHF patients showed that those in the highest ET-1 tertile exhibited more pronounced peripheral congestion, lower urinary sodium levels, and required higher doses of intravenous furosemide, which were associated with an increased one-year mortality risk [27]. Additionally, urinary sodium concentration and its interaction with eGFR are critical for patient profiling in AHF, as low urinary sodium excretion combined with impaired eGFR has been linked to poorer clinical outcomes [28]. Worsening renal function has consistently been identified as a predictor of increased mortality risk in heart failure, with Scr, BUN, and eGFR serving as critical biomarkers of renal clearance capacity. In the present study, significant renal impairment was observed in patients with DR, characterized by markedly higher Cr levels and lower eGFR. Furthermore, the DR group exhibited significantly elevated ALT levels. While some studies suggest that low ALT may reflect frailty in chronic populations, elevated ALT in the context of acute or decompensated heart failure is often associated with hepatic congestion and “cardio-hepatic” syndrome [29]. This elevation reflects increased systemic venous pressure being transmitted to the hepatic sinusoids. Our findings suggest that hepatic congestion may play a mechanistic role in diuretic resistance: the resulting increase in intra-abdominal and renal venous pressure (as evidenced by the significantly higher TRPG in the DR group) reduces the trans-renal pressure gradient, thereby impairing the delivery of loop diuretics to the nephron and promoting a state of resistance.

Left ventricular diastolic dysfunction, a key feature of CHF-related cardiac impairment, contributes to diuretic resistance. Our DR group showed characteristic diastolic dysfunction indicators: higher E/A ratio (abnormal mitral inflow), lower lateral e’ velocity (impaired myocardial relaxation), and elevated LAVI (chronic left atrial remodeling). These abnormalities hinder ventricular filling, raise left atrial pressure, and exacerbate pulmonary congestion and fluid retention—core drivers of diuretic resistance. Evaluating diastolic function in CHF patients with diuretic resistance is crucial, as targeting these deficits may enhance diuretic responsiveness.

However, this study has several limitations due to its small sample size and retrospective design. To obtain more reliable and generalizable results, large-scale, prospective, multi-center clinical studies are warranted. Additionally, different laboratories may employ varying detection methods and reagent kits when measuring serum IL-6, IL-17, and sST2 levels, which could introduce discrepancies in the results and affect the stability and consistency of the predictive model. Therefore, standardization of testing methods is necessary to ensure the accuracy and comparability of findings. Furthermore, the follow-up duration in this study may have been insufficient to fully evaluate the long-term predictive value of serum IL-6, IL-17, and sST2 for diuretic resistance. Extended follow-up periods could provide deeper insights into the association between these biomarkers and diuretic resistance, thereby facilitating further refinement and validation of the predictive model.

## Conclusion

In conclusion, our study demonstrates that elevated serum levels of IL-6, IL-17, and sST2 are independent predictors of diuretic resistance in patients with chronic heart failure. While individual biomarkers show moderate predictive value, their combined assessment significantly enhances diagnostic accuracy, outperforming any single marker. These findings underscore the critical role of systemic inflammation and “cardio-hepatic-renal” interplay in the pathophysiology of diuretic resistance. Clinically, the integration of these inflammatory biomarkers into routine assessment may facilitate early risk stratification and the formulation of individualized diuretic strategies for high-risk CHF patients. Future prospective, multi-center trials are warranted to validate these results and explore the therapeutic potential of targeting these inflammatory pathways.

## Supplementary Information

Supplementary Material 1.

## Data Availability

The data that support the findings of this study are available from the corresponding author upon reasonable request.
